# Le POSSUM: un bon score pour prédire la mortalité du sujet âgé opéré en urgence?

**DOI:** 10.11604/pamj.2016.24.166.9528

**Published:** 2016-06-28

**Authors:** Zeineb Mzoughi, Rached Bayar, Achref Djebbi, Ghofrane Talbi, Hayfa Romdhane, Wafa Aloui, Gharbi Lassaad, Mohamed Taher Khalfallah

**Affiliations:** 1 Université de Tunis El Manar, Faculté de Médecine de Tunis, 1007, Tunis, Tunisie; 2 Service de Chirurgie Viscérale CHU Mongi Slim, Sidi Daoued La Marsa, Tunisie; 3 Service de Gastroentérologie CHU Mongi Slim, Sidi Daoued La Marsa, Tunisie

**Keywords:** POSSUM, score, âgés, urgence, chirurgie, mortalité, POSSUM, score, elderly, emergency, surgery, mortality

## Abstract

**Introduction:**

Le POSSUM (Physiologic and Operative Severity Score for the enUmeration of Mortality and morbidity) est un score prédictif de mortalité qui est largement utilisé en chirurgie aortique élective et abdominale. Le but de notre étude est une validation du POSSUM chez le sujet âgé (>70 ans) opéré pour une urgence digestive. Nous nous proposons d'étudier les meilleurs seuils du POSSUM, composé d'un score physiologique et d'un score opératoire, pour prédire la mortalité dans cette population.

**Méthodes:**

Il s'agit d'une étude rétrospective analytique de type cas témoin à partir d'une série de 291 patients d'âge ≥ 70 ans opérés pour une urgence digestive. Ces patients étaient répartis en deux groupes comportant 50 patients chacun. Le groupe "DC": patients décédés en post opératoire immédiat et le Groupe témoin "SURV" choisis par tirage au sort. Nous avons analysé la fiabilité du POSSUM pour prédire la mortalité et la morbidité. Par la suite, nous avons établi des courbes de ROC pour définir les seuils qui donnent le meilleur couple sensibilité/spécificité.

**Résultats:**

Le score physiologique, le score opératoire et les taux de morbidité et mortalité prédits par POSSUM et la mortalité prédit par P-POSSUM représentaient des facteurs prédictifs de mortalité (P <0,0001). Les valeurs seuils du score physiologique et du score opératoire qui donnent le meilleur couple sensibilité/spécificité, étaient respectivement de 23 et 15.

**Conclusion:**

Prédire la mortalité permet de cibler la prise en charge et d'informer le patient et sa famille des risques encourus.

## Abstract (en)

**Introduction:**

POSSUM (Physiologic and Operative Severity Score for the enumeration of Mortality and morbidity) is a scoring system for predicting mortality which is largely used in elective aortic and abdominal surgery. The aim of our study was to validate POSSUM with regards to elderly patients (> 70 years) undergoing digestive emergency surgery. We wanted to determine the optimal threshold for POSSUM, consisting of a physiological score and of an operative score for predicting mortality in this population.

**Methods:**

This is a case-control analytic retrospective study of 291 patients aged ≥70 years undergoing digestive emergency surgery. These patients were divided into two groups each comprising 50 patients. Group "DC": patients died in the immediate postoperative period and Control group "SURV" chosen by lot. We analyzed the reliability of POSSUM in predicting mortality and morbidity. Subsequently, we created ROC curve to find the thresholds with the best sensitivity/specificity couple.

**Results:**

The physiological score, operative score and mortality and morbidity rates predicted by POSSUM and mortality predicted by P-POSSUM are predictors of mortality (P < 0.0001). The threshold values for the physiological and operative score with the best sensitivity/specificity couple were 23 and 15 respectively.

**Conclusion:**

Predicting mortality in patients allows to target care management programs and to inform the patient and his family of the risks.

## Introduction

Le POSSUM (Physiologic andOperative Severity Score for the enUmeration of Mortality and morbidity) est un score prédictif de mortalité qui a été largement utilisé en chirurgie aortique et abdominale élective [1, 2]. Ce score serait un bon facteur prédictif de mortalité à partir d'un certain seuil dans différentes situations chirurgicales (chez le cirrhotique [3], en urgence [4]). Le but de notre étude est une validation du score POSSUM chez le sujet d'âge avancé (>70ans) opéré dans un contexte d'urgence digestive. Nous nous proposons également d'étudier les meilleurs seuils de ce score pour prédire la mortalité dans cette population.

## Méthodes

il s'agit d'une étude rétrospective type cas témoin d'une série de 291 patients âgés de 70ans et plus opérés pour une urgence abdominale non traumatique entre le 1^er^ Janvier 2008 et le 31 Décembre 2013. Ces patients étaient répartis en deux groupes. Le Groupe "DC" comportant 50 patients décédés en postopératoire immédiat (30 jours post opératoires) et le Groupe témoin "SURV" comportant 50 Patients choisis par tirage au sort parmi tous les autres malades appartenant à la même catégorie d'âge. Nous avons calculé pour chaque patient le score POSSUM et P-POSSUM (Portsmouth-POSSUM). Le système de cotation POSSUM comporte deux composantes. Un score Physiologique (SP) et un Score Opératoire (SO). Le SP est basé sur 12 paramètres physiologiques pour évaluer l´état d´un patient en pré opératoire (Tableau 1), alors que le SO est basé sur 6 paramètres évaluant la complexité de l'intervention chirurgicale (Tableau 2). Les scores physiologiques et opératoires sont combinés avec une analyse en régression logistique pour se transformer en mortalité et morbidité prédites selon les formules suivantes: ln (R/1-R)=7,04+ (0,13xSP) + (0,16xSO) où R représente le taux de mortalité prédit. ln (R'/1-R')=-5,91+ (0,16xSP) +0,19xSO ou R' représente le taux de morbidité prédit. P-POSSUM est établi selon les mêmes systèmes de cotation mais utilise l´analyse linéaire au lieu de l´analyse exponentielle. Pour comparer les 2 groupes, un seuil arbitraire de 25 pour le score physiologique et un seuil à 14 pour le score opératoire étaient initialement fixés. Nous avons analysé au moyen du test de chi-deux de Pearson et du test exact de Fisher la fiabilité du POSSUM pour prédire la mortalité et la morbidité. La valeur pronostique du SP et SO a été étudiée en dégageant les seuils permettant d'obtenir la meilleure sensibilité de ces systèmes de cotation. Nous avons établi des courbes ROC (Receiver Operating Characteristics) pour chercher la valeur de la variable qui correspond au meilleur couple sensibilité-spécificité, après avoir vérifié que l'aire sous la courbe était significativement > 0,500.

**Tableau 1:** POSSUM score physiologique (SP)

	1	2	4	8
Age	≤60	61-70	≥71	
Score de Glasgow	15	12-14	9-11	≤8
Signes respiratoires	Pas de dyspnée	Dyspnée légère/ signe minime de BPCO*	Dyspnée invalidante/ signe modéré BPCO*	Dyspnée importante / fibrose
Signes cardiaques	Pas de signe d’insuffisance cardiaque	Traitement diurétique, digitalique, anti angineux ou anti HTA**	Traitement par antivitamine K	Turgescence jugulaire, cardiomégalie
Electro-cardiogramme	normal	Fibrillation atriale + rythme =60-90		Tout autre rythme anormal
Fréquence cardiaque (bpm)	50-80	<40-49 Et 81-100	101-120	<39 et >120
Pression artérielle systolique (mmHg)	110-130	100-109 et 131-170	90-99 >171	<89
Hémoglobine (g/dL)	13-16	11,5-12,9 et 16,1-17	10-11,4 et 17,1-18	<9 et >18,1
Leucocytes (/mm3)	4000-10000	3100-3999 et 10100-20000	<3000	>20100
Natrémie (mEql/L)	>136	131-135	126-130	<125
Kaliémie (mEql/L)	3,5-5	3,2-3,4 et 5,1-5,3	2,9-3,1 et 5,4-5,9	<2,8 et>6
Urée (mg/L)	<0,45	0,46-0,6	0,61-0,90	>0,90

* BPCO =Bronchopneumopathie chronique obstructive

** Hypertension artérielle

**Tableau 2:** POSSUM score opératoire

	1	2	4	8
Gravité d’intervention	Mineure	Moyenne	Majeure	Majeure++
Nombre d’intervention	1		2	>2
Pertes sanguines	<100	100-500	500-1000	>1000
Contamination péritonéale	Aucune	Mineure	Collections purulentes	Diffusé
Cancer	Aucun	Localisé	Métastases ganglionnaires	Métastase(s) à distance
Circonstance d’intervention	Réglée		Urgence >24h mais <24H	Sans délai

Chirurgie moyenne: cholécystectomie, appendicectomie, mastectomie, RTU prostate. Chirurgie majeure: toute laparotomie, résection du tube digestif, cholécystectomie avec cholédocotomie, chirurgie vasculaire périphérique ou amputation majeure. Chirurgie majeure +: toutes interventions sur l'aorte, amputation abdominopérinéale, résection pancréatique ou hépatique, œsophago-gastrectomie.

## Résultats

**Population étudiée:** L'âge moyen des patients était 77,06 ans dans le groupe "DC" contre 76,24 ans pour le groupe "SURV". Le sex ratio dans le groupe "DC" était à 1,27. Il était à 1,17 dans le groupe "SURV". Soixante-douze pourcent des patients de chaque groupe avaient au moins une comorbidité. Les diagnostics préopératoires étaient répartis en 3 groupes: les urgences hépatobiliaires, les urgences néoplasiques et les autres urgences. Les urgences hépatobiliaires représentaient 30% pour le groupe "DC" et 18% pour le groupe "SURV". Les pathologies néoplasiques représentaient 8% des diagnostics préopératoires pour le groupe "DC" et 8% des diagnostics pour le groupe "SURV". Les autres diagnostics représentaient 62% des cas pour le groupe "DC" et 73% des cas dans le groupe "SURV". La répartition des gestes opératoires réalisés en urgence est résumée dans le Tableau 3.

**Tableau 3:** Geste opératoire réalisé en urgence

Type d’intervention	DC	SURV
Toilette péritonéale	31(62)	36(72)
Cholécystectomie	9(18)	8(16)
Cholédocotomie et extraction de calcul	5(10)	1(2)
Appendicectomie	3(6)	8(16)
Résection intestinale	16(32)	13(26)
Suture d’ulcère	3(6)	6(12)
Mise à plat d’un abcès	7(14)	5(10)
Cure hernie/éventration	1(2)	4(8)
Abstention thérapeutique	3(6)	1(2)
Stomie	14(28)	13(26)
Drainage	15(30)	18(36)

**DC :** groupe décédés, **SURV :** groupe témoin

**Figure 1:**
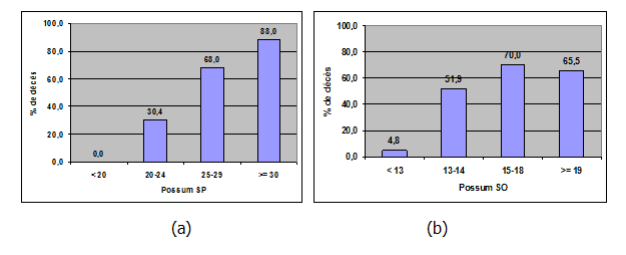
Sensibilité du score Possum pour prédire la mortalité; (a): score physiologique (SP); (b): score opératoire (SO)

**Figure 2:**
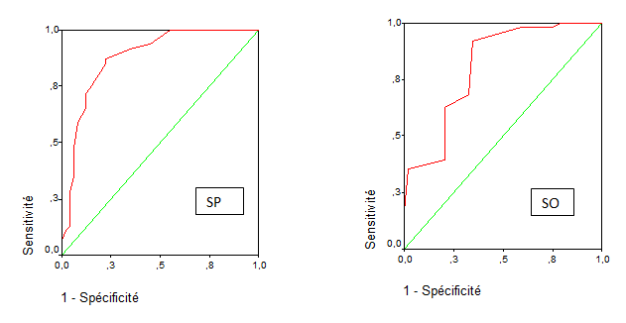
Courbes de ROC (a): score physiologique: le point culminant est situé à 23; (b): score opératoire: le point culminant est situé à 15

**Valeur pronostique du POSSUM et seuils:** Le SP moyen dans le groupe "DC" était de 30,7 contre 21,1 pour le groupe témoin. Le SO moyen était de 17,9 versus 13,6. Le taux de mortalité prédit par POSSUM était plus élevé dans le groupe "DC" 45,8% versus 14,1%. Pour le score P-POSSUM, le taux de mortalité prédit du groupe "DC" était également plus élevé: 29,8% versus 6,3%. Le taux de morbidité prédit était de 86,3% pour le groupe "DC" alors qu'il était de 50,4% des cas pour le groupe témoin. Un SP > 25 et un SO> 14, les taux de morbidité et mortalité prédits par POSSUM et la mortalité prédit par P-POSSUM représentaient des facteurs significatifs de mortalité chez le sujet âgé opéré pour une urgence digestive (p<0,0001). La mortalité était nulle pour un SP < 20. Pour un SP ≥ 30, la sensibilité pour prédire la mortalité était de 88%. Pour un seuil ≥ 28, cette sensibilité diminuait à 83,3%. Concernant le SO, intégrant les données opératoires, une valeur ≤ 13 s'accompagnait d'un taux de décès prédit de 4,8%. La sensibilité de la valeur ≥15 était de 70%. On note que des valeurs plus importantes du score ≥19 diminuent cette sensibilité (65,5%). La valeur seuil ≥18 a la meilleure sensibilité (69,7%) pour prédire la mortalité (Figure 1). Le point d'inflexion de la courbe de ROC traduisant le meilleur couple sensibilité /spécificité se situait au seuil 23 pour le SP. Pour le SO, ce point se situait au seuil de 15 (Figure 2).

**Valeur pronostique du POSSUM et seuils:** Le SP moyen dans le groupe "DC" était de 30,7 contre 21,1 pour le groupe témoin. Le SO moyen était de 17,9 versus 13,6. Le taux de mortalité prédit par POSSUM était plus élevé dans le groupe "DC" 45,8% versus 14,1%. Pour le score P-POSSUM, le taux de mortalité prédit du groupe "DC" était également plus élevé: 29,8% versus 6,3%. Le taux de morbidité prédit était de 86,3% pour le groupe "DC" alors qu'il était de 50,4% des cas pour le groupe témoin. Un SP > 25 et un SO> 14, les taux de morbidité et mortalité prédits par POSSUM et la mortalité prédit par P-POSSUM représentaient des facteurs significatifs de mortalité chez le sujet âgé opéré pour une urgence digestive (p<0,0001). La mortalité était nulle pour un SP < 20. Pour un SP ≥ 30, la sensibilité pour prédire la mortalité était de 88%. Pour un seuil ≥ 28, cette sensibilité diminuait à 83,3%. Concernant le SO, intégrant les données opératoires, une valeur ≤ 13 s'accompagnait d'un taux de décès prédit de 4,8%. La sensibilité de la valeur ≥15 était de 70%. On note que des valeurs plus importantes du score ≥19 diminuent cette sensibilité (65,5%). La valeur seuil ≥18 a la meilleure sensibilité (69,7%) pour prédire la mortalité (Figure 1). Le point d'inflexion de la courbe de ROC traduisant le meilleur couple sensibilité /spécificité se situait au seuil 23 pour le SP. Pour le SO, ce point se situait au seuil de 15 (Figure 2).

## Discussion

Dans notre étude, nous avons validé le score de POSSUM comme étant prédictif de mortalité chez le sujet âgé de plus de 70 ans opéré pour une urgence abdominale. Toutes les données à savoir un SP, SO supérieurs aux seuils définis, ainsi que la morbi-mortalité prédite étaient significativement plus élevés dans le groupe décédé (p<0,0001). Par ailleurs, les seuils pour avoir la meilleure sensibilité étaient de 30 et 18 respectivement pour le SP et SO. Ces seuils sont proches des seuils choisis arbitrairement dans la première partie de l'étude. La valeur seuil pour avoir le meilleur couple Se/Sp était 23 pour SP et 15 pour SO. Bien que rétrospective, notre étude nous a permis de calculer le POSSUM à partir des observations des patients. Les données de ce score semblent donc anodines et faciles à collecter. Le caractère analytique et l'utilisation du cas témoin ne peut que conférer de la crédibilité aux résultats. Le tirage au sort du groupe témoin constitue également une force de cette analyse puisque les différences entre les deux groupes étaient purement dues au hasard y compris dans les valeurs du POSSUM. Les limites de notre étude étaient essentiellement représentées par l'absence de suivi au-delà des 30 jours post opératoires immédiats. Nous n'avons pas de données concernant l'évolution des survivants. L'évaluation du risque opératoire, et surtout celui de décès post opératoire reste un souci quotidien du chirurgien. Certains auteurs ont constaté, dans un audit de la mortalité chez les personnes âgées, que les chirurgiens ou les anesthésistes ont prédit le décès avec une spécificité de 89 % [5]. Hartley et Sagar ont trouvé que l'avis d'expert était plus sensible que le POSSUM (88% Vs 64%) [6]. Ces dernières années, la subjectivité a laissé place aux évaluations scientifiques analytiques et objectives. Les scores prédictifs ne cessent d'être mis en avant dans différents domaines. De nombreuses études ont largement validé le POSSUM pour évaluer le risque de morbi-mortalité après chirurgie abdominale ou pancréatique [7, 8]. Le POSSUM permettrait en pratique de prédire une surmortalité du sujet âgé permettant d'avoir une balance bénéfice risque dans les interventions non urgentes. Dans un contexte d'urgence, la problématique est différente. Calculer le POSSUM servirait plus à organiser la prise en charge du patient, intensifier la réanimation ou décider de l'opérer dans les plus brefs délais. Il s'agit également d'informer le patient et sa famille du pronostic de la façon la plus objective possible. Dans la majorité des études de mortalité chez le sujet âgé opéré en urgence, le POSSUM ressort comme prédictif de mortalité de façon indépendante [9, 10]. La valeur prédictive du POSSUM a été étudiée dans une population de cirrhotiques opérés en urgence. Dans cette étude prospective, le POSSUM n'avait plus de valeur pour prédire la mortalité dans les stades très avancés de cirrhose [3]. Dans la majorité des études, le seuil choisi des systèmes de cotations SP et SO du score Possum reste arbitraire, comme dans notre série. Dans une série étudiant le POSSUM dans la chirurgie aortique élective, le seuil SP ayant la meilleur Se/Sp était défini par la courbe ROC à 22 [11]. Dans notre série, ce seuil était à 23 avec un seuil du SO à 15. Ces seuils pourraient être utilisés dans de nouvelles études prospectives analysant l'apport du POSSUM dans des situations particulières (sujet âgé, cirrhotique, contexte d'urgence, chirurgie néoplasique…) pour prédire la morbi-mortalité post-opératoire.

## Conclusion

Le POSSUM est un bon score permettant de prédire de façon objective la mortalité du sujet âgé opéré pour une urgence digestive. Son calcul, taxé de complexe, s'est avéré facile à travers des observations de malades. Le POSSUM, chez le sujet âgé dans l'absolu et dans le contexte d'urgence plus particulièrement, est très utile pour cibler la prise en charge. Il permettrait d'identifier les patients nécessitant une réanimation intensive, une intervention dans les plus brefs délais et pour informer le patient et sa famille des risques encourus. Les seuils du SP et SO fixés dans notre étude pourraient servir dans d'autres études prospectives randomisées analysant la mortalité dans différentes situations pathologiques.

### Etat des connaissances actuelle sur le sujet

- Le POSSUM est un score général considéré comme prédictif de mortalité;
- le POSSUM est un facteur prédictif de mortalité après chirurgie.

### Contribution de notre étude à la connaissance

- Le possum permet de prédire de façon fiable la mortalité du sujet âgé opéré d'une urgence abdominale. Il s'agit d'un facteur significatif de mortalité;
- Le seuil de 23 pour le score physiologique et 15 pour le score opératoire ont la meilleure valeur prédictive.
